# Privacy-preserving federated genome-wide association studies via dynamic sampling

**DOI:** 10.1093/bioinformatics/btad639

**Published:** 2023-10-19

**Authors:** Xinyue Wang, Leonard Dervishi, Wentao Li, Erman Ayday, Xiaoqian Jiang, Jaideep Vaidya

**Affiliations:** Management Science and Information Systems Department, Rutgers University, New Brunswick, NJ 07102, United States; Department of Computer and Data Sciences, Cleveland, OH 44106, United States; Department of Health Data Science and Artificial Intelligence, Houston, TX 77030, United States; Department of Computer and Data Sciences, Cleveland, OH 44106, United States; Department of Health Data Science and Artificial Intelligence, Houston, TX 77030, United States; Management Science and Information Systems Department, Rutgers University, New Brunswick, NJ 07102, United States

## Abstract

**Motivation:**

Genome-wide association studies (GWAS) benefit from the increasing availability of genomic data and cross-institution collaborations. However, sharing data across institutional boundaries jeopardizes medical data confidentiality and patient privacy. While modern cryptographic techniques provide formal secure guarantees, the substantial communication and computational overheads hinder the practical application of large-scale collaborative GWAS.

**Results:**

This work introduces an efficient framework for conducting collaborative GWAS on distributed datasets, maintaining data privacy without compromising the accuracy of the results. We propose a novel two-step strategy aimed at reducing communication and computational overheads, and we employ iterative and sampling techniques to ensure accurate results. We instantiate our approach using logistic regression, a commonly used statistical method for identifying associations between genetic markers and the phenotype of interest. We evaluate our proposed methods using two real genomic datasets and demonstrate their robustness in the presence of between-study heterogeneity and skewed phenotype distributions using a variety of experimental settings. The empirical results show the efficiency and applicability of the proposed method and the promise for its application for large-scale collaborative GWAS.

**Availability and implementation:**

The source code and data are available at https://github.com/amioamo/TDS.

## 1 Introduction

Over the last few years, genome-wide association studies (GWAS) have allowed a significant understanding of the associations between complex genetic variants and traits in individuals (Rietveld *et al.* 2013, Buniello *et al.* 2019).

The efficacy of GWAS in detecting rare yet crucial genetic signals is influenced by various factors, such as inherent associations between genetic variants and traits (Goldstein 2009), data quality (Anderson *et al.* 2010), and sample size (Lander, 2011). Collaborative GWAS, which entails the joint analysis of multiple datasets, has demonstrated an enhanced ability to detect rare variations (Lango Allen *et al.* 2010, Lander 2011). However, privacy concerns that deter individuals and institutions from sharing private data (Gymrek *et al.* 2013, Harmanci and Gerstein 2016) significantly hinder collaborative studies. Moreover, legislative policies and regulations restricting the sharing of sensitive genetic data (Act 1996, Voigt and Von dem Bussche 2017) further limit the prevalence of collaborative GWAS.

Privacy-aware collaborative GWAS frameworks have been developed, leveraging modern cryptographic and machine learning techniques, such as Homomorphic Encryption (HE), Secure Multi-Party Computation (SMC), and Federated Learning (FL). Homomorphic encryption is a cryptographic technique that allows specific operations (e.g. addition and/or multiplication) to be conducted directly on encrypted data. While HE offers robust security, it increases the computation and communication burdens for the researchers. Moreover, HE supports only addition and/or multiplication operations. Nonlinear operations necessary for regression analysis (e.g. logistic regression) can only be approximated, impacting result accuracy (Blatt *et al.* 2020).

SMC-based frameworks enable researchers to perform analyses collectively over securely shared data without direct access to the underlying input. SMC solutions, such as those presented in (Shi *et al.* 2016), often utilize a cryptographic primitive named secret sharing (Shamir 1979), where each researcher generates a secret share of their private data, computes intermediate results from the secret shares, and exchanges them. Each researcher then computes the final results based on all intermediate results. Although the secret shares do not disclose any private information, the computation/communication costs of such solutions often render them impractical for complex tasks. Moreover, if one or more parties are compromised, the privacy of all participating parties is at risk. Techniques such as threshold secret sharing can protect against this, but they substantially increase the share size and the corresponding computation/communication cost (Ito *et al.* 1993).

Federated learning is an alternative machine learning technique that trains a model across multiple parties with local datasets. In FL-based GWAS, each researcher constructs a model with local data and shares the parameters (e.g. gradients) with the server. The server amalgamates the parameters from all parties and redistributes them to all researchers, who then iteratively update the model until it converges. FL-based approaches, for instance (Wu *et al.* 2021), offload the computational overhead from the server to the researchers, increasing communication costs due to the iterative process. Additionally, sharing the intermediate results of the local model may expose private information (Zhu *et al.* 2019).

Recent works have attempted to leverage these techniques to address privacy concerns in collaborative GWAS while tackling computational and communication bottlenecks. Secure GWAS (Cho *et al.* 2018) is a Principal Component Analysis (PCA)-based GWAS protocol based on SMC, using random projections and precomputed values to expedite the process. However, their protocol is communication-intensive, as it requires the entire dataset to be encrypted and distributed among all the parties. In the case of a dataset with 22 536 individuals and 509k array genotypes, the total communication cost approximates 700 gigabytes.

An alternative approach to collaborative GWAS is based on meta-analysis, a statistical tool that combines summary statistics from similar studies and has proven effective in identifying possible associations between SNPs and traits (Speliotes *et al.* 2010, Lander 2011). While meta-analysis requires only the exchange of summary statistics between collaborating parties, it is still possible to identify individuals and their relatives based on these summary statistics (Homer *et al.* 2008). Moreover, results from meta-analysis of heterogeneous cohorts (e.g. sample size, phenotyping, imputation) can be biased (Kanai *et al.* 2022). sPLINK (Nasirigerdeh *et al.* 2022) offers a privacy-aware alternative for meta-analysis in GWAS based on federated learning. In this method, each client computes local parameters and masks them with noise. They then share the noise with the compensator and the noisy local parameters with the server. The compensator aggregates the clients’ noise values and sends the aggregated noise to the server, which computes the global parameters by summing the noisy local parameters and subtracting the aggregated noise. Compared to SMC-based approaches, sPlink is computationally efficient as heavy computations are distributed across clients and can be performed in plaintext. However, intermediate results (e.g. Hessian Matrix) are shared in plaintext, which could lead to information leakage (Zhu *et al.* 2019). Furthermore, a breach of the compensator—an event that has become increasingly probable in this era of frequent sensitive user data leaks—jeopardizes the privacy of all parties involved. Another issue is the slow convergence rate of the federated learning model, which may not be guaranteed in the presence of non-IID data (Zhao *et al.* 2018). In a dataset with 5343 individuals and 600k genotypes, the total analysis using sPlink requires about 75 min, and the total communication costs for 20 iterations are around 11 GB. In conclusion, existing methods are impractical for large datasets, which is crucial for obtaining reliable GWAS results.

In this work, we introduce a new, efficient, privacy-preserving federated GWAS framework named Two-Step Dynamic Sampling (*TDS*) GWAS. Our approach unfolds in two phases. In the first phase, local parties collaboratively identify loci in their local data that are not significantly associated. This phase substantially curbs computation and communication costs by removing a large number of non-significant loci from subsequent analysis. In the second phase, all the local parties iteratively share portions of their private datasets with the server. The server performs GWAS on the pooled data and returns the results to the local parties. Our approach, improving existing federated learning methods and meta-analysis, involves sharing partial data, which allows us to achieve the same results as those obtained from aggregated analysis and enhances the performance in identifying significantly associated loci, even with datasets having between-study heterogeneity and imbalanced phenotype distributions. To manage the privacy risk introduced by sharing partial data, we apply the permutation techniques used in (Dervishi *et al.*^2023^) to keep the privacy risk below a baseline that aligns with the risk associated with sharing summary statistics.

Although our primary focus is logistic regression test, our proposed framework can be generalized to accommodate other useful statistics in GWAS, such as $\chi^{2}$ statistics, linear regression tests, and Cochran–Armitage trend tests (CATT) statistics (Armitage 1955). We assess the proposed framework using two real genomic datasets from the 1000 Genome Project and OpenSNP. We simulate the phenotypes for 2400 individuals in 1000 Genome Project datasets and divide the data between two parties, considering different levels of phenotype distribution imbalance and between-study heterogeneity. Compared to standard meta-analysis, the proposed method consistently delivers higher accuracy rates for identifying significant associations across different scenarios. Thanks to our key two-step approach, *TDS* reduces the overall runtime and is less dependent on powerful hardware than existing cryptographic approaches. Our work showcases a highly effective and efficient method for conducting privacy-preserving collaborative GWAS.

## 2 Materials and methods

### 2.1 Logistic regression

Our study concentrates on case-control GWAS. In this method, the genomes of individuals exhibiting a particular trait or phenotype (the case group) are compared to those lacking that trait (the control group). The GWAS outcome comprises the SNPs most strongly associated with the studied trait. In such study, the phenotype of individual *i* is denoted as $p_{i}\in 0,1$ and genotypes as $x_{i}\in 0,1,2^{n}$. Here, 0, 1, or 2 represent the number of minor alleles in *n* different SNPs of individual *i*. For ease of distinction, we utilize bold and regular symbols to represent vector/matrix and scalar variables, respectively. We employ logistic regression to conduct the single-SNP analysis, testing one SNP at a time and producing a *P*-value for each SNP. Specifically, the probability of individual *i* having the disease, conditioned on his/her genotype *x_i_*, is expressed as


$$P(p_{i}=1|x_{i},\beta )=\frac{1}{1+\text{exp}(-\beta x_{i}^{j})},$$

where *x_i_* is the genotype of a SNP for individual *i*, and *β* represents the genetic effect. We utilize the standard Wald test (Bewick *et al.* 2005) to test the null hypothesis that the SNP has no effect ($H_{0}:\beta =0$) against the alternative hypothesis that it does impact the phenotype ($H_{0}:\beta \neq 0$). We reject the null hypothesis and deem a statistical test significant if the *P*-value of the Wald test statistics falls below a predefined significance threshold *μ*. A typical GWAS simultaneously tests millions of SNPs. The Bonferroni correction (Bonferroni 1936) is commonly used to adjust the threshold and control the family-wise error rate (i.e. the probability of making at least one false positive). For *n* SNPs tested in one study, the adjusted significance threshold is defined as α/n, where *α* is the target significance level (e.g. 0.05).

### 2.2 Two-step dynamic sampling (TDS) framework

In the proposed framework, a group of researchers possesses their private genotype arrays and phenotype vectors. They aim to collaboratively perform a logistic regression test to identify genetic markers (i.e. SNPs) significantly associated with the phenotype by sharing partial data generated from their local dataset with a third party (e.g. a server) equipped with extensive computational resources. Upon receipt of this shared data, the server performs the necessary computations and returns the association results (i.e. *P*-values) to the researchers. The proposed framework assures robust results despite between-study heterogeneity through an iterative process to share the partial data, allowing efficient computation while safeguarding the privacy of the participant individuals.

**Figure 1.**
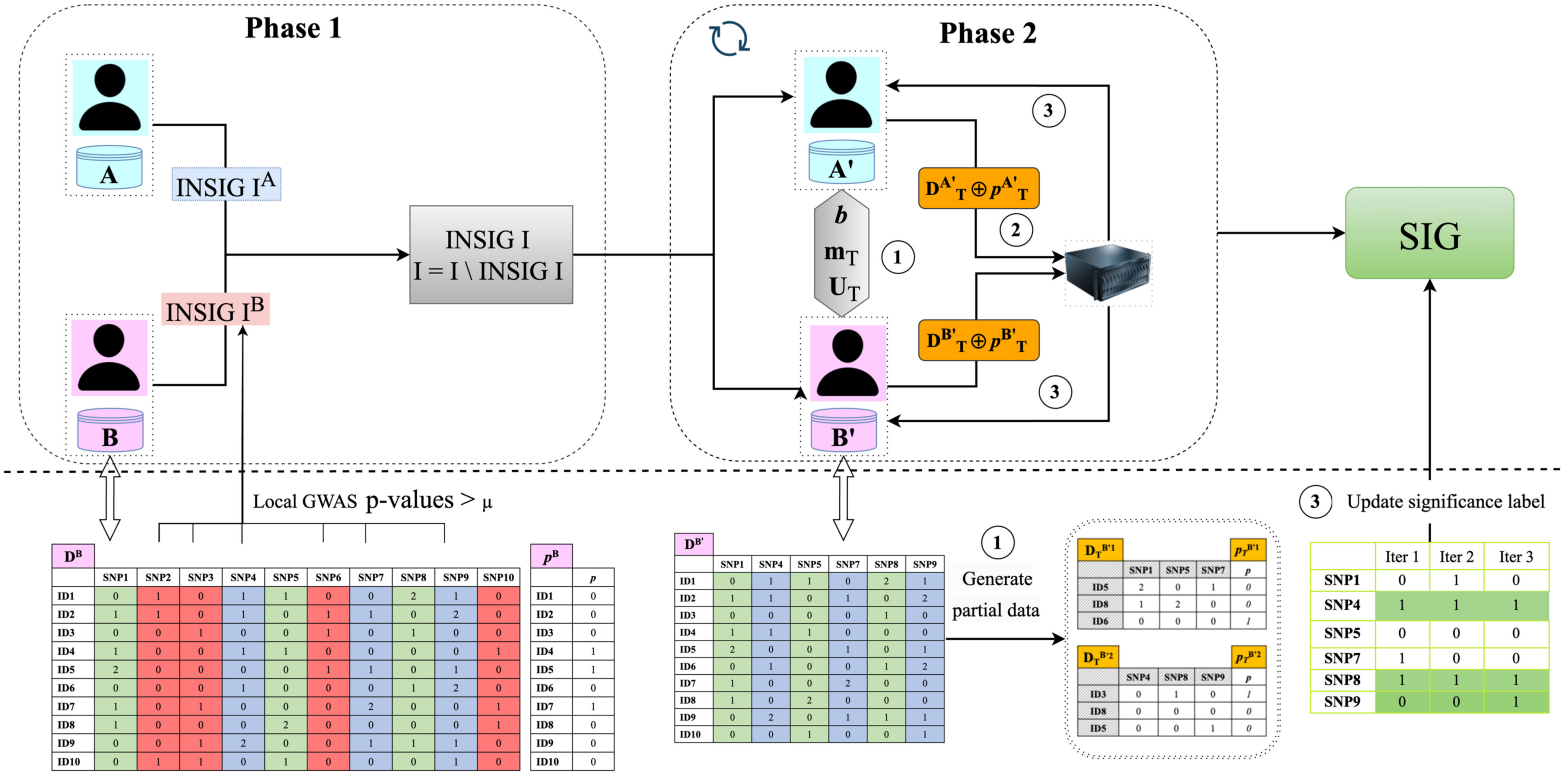
Workflow overview of the proposed framework. In *Phase 1*, researchers A and B perform GWAS on their local genotype arrays and phenotype vector. They share the IDs of insignificant SNPs (red and blue columns) to obtain the intersection of the shared IDs. Both researchers then remove the insignificant SNPs in both datasets (red columns). *Phase 1* consists of two stages. Synchronization stage [(1) in the figure]: Two researchers initially decide whether they will continue the process; if they continue, they decide on the partition of SNP set (***b***), number of SNPs $m_{T}$, and the common permutation seed $U_{T}$ for each batch. Here, the entire set of SNPs (*I*) is partitioned into two batches for the illustration. Each researcher then uniformly samples *K* individuals to generate the partial data, which is later sent to the server. The grayed cells in the partial data (e.g. $D_{1}^{B\prime }$) suggest that the SNP IDs and individual IDs are removed from the genotype arrays and phenotype vectors before outsourcing. Outsourcing stage: In (2), A and B send the shuffled and anonymous partial data to the server; In (3), the server performs logistic regression tests and returns the *P*-values to both researchers. Each researcher updates the significance level of each SNP based on *P*-values obtained from previous iterations and threshold *μ*.

This section introduces the Two-step Dynamic Sampling (*TDS*) federated GWAS framework. The proposed framework comprises two phases. In *Phase 1*, both researchers conduct the computation locally and collaboratively identify the insignificant associations. In *Phase 1*, both researchers iteratively share the partial data, extracted from their local datasets, with the server to detect significant associations for the remaining SNPs. For the simplicity of exposition, we consider only two researchers *A* and *B*, but our proposed approach can readily be extended to accommodate multiple researchers. We assume that the researchers are honest and the server is honest-but-curious in the proposed design. An overview of the proposed framework is depicted in Fig. 1 and Supplementary Material S1, with the details of the two phases discussed in the subsequent sections.

#### 2.2.1 *Phase 1*: Detecting insignificant associations from local data

Let *S* denote the server and $D^{r}\in {\left\{0,1,2\right\}}^{n*N^{r}}$ represent the original dataset of researcher r∈A,B, where *n* indicates the number of SNPs and *N^r^* denotes the number of individuals. We denote the set of SNP IDs as $I=\{SNP_{1},SNP_{2},\ldots ,SNP_{n}\}$ and the phenotype vector of each researcher as $p^{r}\in {\left\{0,1\right\}}^{N^{r}}$.

In *Phase 1*, both researchers collaborate to identify insignificant associations. Initially, each researcher conducts computation with their local dataset and determines the *P*-values for each SNP in *I*. Utilizing the selected threshold *μ*, each researcher decides the significance conditions for all the SNPs in *I*. More precisely, an association is deemed insignificant if its *P*-value exceeds *μ*.

To simplify the illustration, we assume both researchers choose identical thresholds. However, they may independently select different thresholds. Subsequently, they share the set of insignificant SNP IDs, $\mathrm{INSIG}\;I^{r}$, with each other to identify SNPs deemed insignificant in both datasets. This process is straightforward when both researchers are honest. For example, researcher A can send $\mathrm{INSIG}\;I^{A}$ to B, who then returns $\mathrm{INSIG}\;I=\mathrm{INSIG}\;I^{A}\cap \mathrm{INSIG}\;I^{B}$, i.e. the SNP IDs deemed insignificant in both datasets, to A. In scenarios with multiple researchers, one can serve as the aggregator of all SNP ID lists from others. After identifying mutually insignificant SNPs, each researcher removes data associated with the SNPs in INSIG I from their datasets. The computation of SNP intersection is efficient and demands minimal computational and communication costs from the researchers. The computational complexity of executing the logistic regression test using local datasets depends on the dataset size and the algorithms employed. For example, the training time complexity of a standard binary logistic regression is $O(n*N^{r})$.

While the primary objective of *Phase 1* is to detect insignificant associations, it is crucial to note that researchers do not seek to identify all insignificant associations at this stage. In other words, both researchers aim to detect the easily identifiable insignificant associations, thereby reducing the number of SNPs to be examined in the subsequent phase. An implicit requirement is to avoid erroneously including significant SNPs in INSIG I (i.e. false negatives). Given this consideration, we employ larger *P*-value thresholds (e.g. μ=0.3) since the Bonferroni correction can be overly conservative, leading to a significant number of false negatives (Chen *et al.* 2021, Gumpinger *et al.* 2021).

#### 2.2.2 Phase 2: Detecting federated significant associations via dynamic sampling

In this phase, both researchers iteratively send data related to the remaining SNPs (I∖INSIG I) to the server, facilitating the computation of logistic regression. Each iteration encompasses two stages: synchronization and outsourcing.

At iteration *T*, during the synchronization stage, following a similar approach to *PPKI* (Dervishi *et al.* 2023), researchers collaboratively decide on the set of SNPs, i.e. $I_{T}=SNP_{1},SNP_{2},\ldots ,SNP_{m_{T}}$, to share with the server and a common seed *U_T_*. Here, $I_{T}\subseteq I\setminus \mathrm{INSIG}\;I$. The common seed *U_T_* enables researchers to shuffle their shared SNPs in the same way, ensuring the correctness of computation results while preventing the server from inferring the actual genome sequence of individuals.

This process can be extended to a batch-wise approach, as depicted in Fig. 1. For remaining SNPs post *Phase 1*, researchers can cooperatively partition the SNP IDs into *b* non-overlapping subsets, i.e. $I\setminus \mathrm{INSIG}\;I=I_{1,T}\cup I_{2,T}\cup \cdots \cup I_{b,T}$ and $I_{i,T}\cap I_{j,T}=\emptyset$ for i≠j, and decide the common seed vector $U_{T}$ for *b* batches. As this step does not involve the server, one researcher could determine the SNPs IDs and the seed and broadcast them to other researchers. Researchers share the partial data derived from the *b* batches (elaborated further later) with the server to simultaneously compute univariate test statistics of all the SNPs. While this approach reduces the communication load, it could raise the privacy risk, particularly when each batch contains a few individuals. We argue that researchers only need to share data of some batches after a few iterations as they acquire high confidence in most SNPs (as demonstrated in the experiment results in Section 4). We also employ the technique in *PPKI* (Dervishi *et al.* 2023) to control the privacy leakage and scrutinize the privacy risk in Section 3.

Next, both researchers sample *K* individuals uniformly at random from their local datasets (without maintaining the case/control balance to avoid oversampling specific individuals when there is severe between-study heterogeneity). Even though it might seem counter-intuitive, using a random subset suffices for good results and concurrently reduces the privacy risk. The researchers shuffle the SNPs (i.e. columns) according to the common seed $U_{T}$ with the selected individuals. Each researcher *R^i^* then shuffles the individuals (i.e. rows) and removes the SNP identifier and sample identifier from the genotype arrays and phenotype vector. Notably, the permutation of individuals can be done independently and is unknown to the other researcher(s).

In this scenario, the number of SNPs in each batch ($m_{b,T}$) and the number of selected individuals (*K*) are system parameters to control the computational load and, more importantly, the computation accuracy and privacy. The effect of $m_{b,T}$ on privacy risk has been extensively examined (Dervishi *et al.* 2023). In this article, we focus on the impact of *K* on performance and use *m *=* *300 as the default value [a small number, e.g. 250, is suggested (Dervishi *et al.* 2023) to limit the privacy risk and restrict the server’s ability to un-shuffle the SNP sequences].

During the outsourcing stage, both researchers transmit the shuffled genotype arrays and phenotype vector, ${\tilde{D}}_{T}⊕{\tilde{p}}_{T}$, to the server (here, $\overset{˜\cdot }{\cdot }$ implies that the data is anonymized by removing identifiers, and ⊕ is the matrix concatenation operator). The server then conducts the computation on pooled data and returns the *P*-values of each association test to both researchers. Each researcher reverts the order, compares the *P*-values with the thresholds (*μ*), and obtains the significance condition of all the SNPs in I∖INSIG I. For example, if the returned *P*-value of *SNP_i_* is greater than *μ*, then its significance label, denoted as $l_{i,T}$, is set to be 0, indicating the association between $SNP_{i}$ and the phenotype is insignificant. Each researcher then uses the majority voting strategy to update the significance label based on current and previous significance labels. Given a list of significance labels from *T* iterations, $l_{1},l_{2},\ldots ,l_{T}$, the significance label $l^{*}$ is given as the label that appears most often, i.e. $l^{*}=\mathrm{MODE}(l_{1},l_{2},\ldots ,l_{T})$. Note that the thresholds *μ* can differ from the one used in *Phase 1*, as researchers can select more stringent values to lower the false negative rates. In Section 4, we empirically demonstrated the impact of *μ* on the utility. In the next synchronization phase, the researchers need to determine whether to continue. Here, we consider a simple condition where if the significance label for all the SNPs remains constant, both researchers decide to halt the iteration.

*Phase 1* offloads the computational burden to the server, thereby reducing inter-site communication and ensuring only necessary information is exchanged. The computation cost for the researchers is incurred when performing sampling and shuffling, a task which only needs execution once. On the server side, the computation complexity hinges on the statistical test implemented, the batch size received per iteration, and the overall number of iterations. With *T* iterations, the computation complexity for the server becomes O(TK*m). Notably, since all logistic regression computations are conducted in plaintext (applicable to *Phase 1* as well), both the researchers and the server can harness parallel computation techniques to expedite the process (Sikorska *et al.* 2013). The main communication bottleneck arises from sharing the partial data. For a SNP encoded with 2 bits, a single iteration sharing *m* SNPs of *K* individuals necessitates 2*mK* bits in a simplistic scenario. To reduce the size and accelerate the transmission process, efficient compression schemes such as 7zip can be deployed. It is important to note that all the steps above can be easily generalized to the case where there are multiple researchers, since all the communications are between the server and the researcher(s).

## 3 Privacy analysis

This study encompasses multiple researchers aiming to conduct collaborative logistic regression tests by sharing partial data extracted from their datasets with a server possessing substantial computational resources. Upon receiving this data, the server performs necessary computations and returns the association results (in terms of *P*-values) to both researchers. The objective of each researcher is to discern significant associations between SNPs and specific phenotype vectors across federated datasets, all while maintaining participant privacy.

Genomic data sharing is vulnerable to several known privacy attacks, including membership inference attacks (Homer *et al.* 2008, Wang *et al.* 2009), attribute inference attacks (Humbert *et al.* 2013), and reconstruction attacks (Mizas *et al.* 2008). Typically, adversaries are assumed to have access to the target’s full or partial genomic sequences, exploiting side information to increase the power of their attacks. This study focuses on general privacy risks without specifying any particular attacks, aiming to mitigate privacy risks associated with iterative partial data sharing. *TDS* presumes an honest-but-curious server and legitimate researchers, a threat model widely accepted within the medical informatics context. Therefore, during *Phase 1*, the two researchers directly exchange *INSIG I*, which leaks a little bit of extra information (i.e. the SNPs that are insignificant for the other researcher.) If this leakage is not acceptable, the researchers can employ Private Set Intersection algorithms, such as (Pinkas *et al.* 2014) or (Chen *et al.* 2018), to obtain the *INSIG I* without revealing real SNP IDs to each other.

Next, we discuss the potential privacy risks in *Phase 2*. Suppose the server possesses a target and that target’s partial genome sequence. While the server adheres to the protocol and performs computations accurately, it may attempt to infer additional information—specifically, the genome sequences of the target. To implement subsequent attacks, such as membership inference and reconstruction attacks, the server attempts to match the target’s sequence with the shared partial data from both researchers. This is achievable if (i) the server can match the unordered SNP sequence shared by the researchers with the target’s SNP sequence in each iteration and (ii) the server can discern the linkage among the SNPs shared throughout the iterative process. Hence, privacy risks depend on the number of shared SNPs, the number of iterations, and the correlations between SNPs.

The system and threat models of *TDS* align with those of *PPKI* (Dervishi *et al.* 2023), a federated framework for kinship relatedness identification, which shares metadata generated from local datasets with the server to facilitate the necessary computations. Two primary differences exist between *TDS* and *PPKI*. First, in *PPKI*, researchers share metadata that comprises shuffled SNPs and SNP IDs (without any correspondence between SNPs and SNP IDs in the metadata). In contrast, *TDS* assumes that SNP IDs are not revealed to the server. Second, *PPKI* employs a non-iterative approach, meaning the synchronization and outsourcing stage is performed only once. We then discuss how *TDS* reduces privacy risks by keeping SNP IDs hidden from the server and mitigates privacy risks introduced by the iterative process.

Below, we review the privacy risks and the membership attack considered in *PPKI*. In *PPKI*, the metadata received by the server includes the set of SNP IDs and shuffled SNPs. Assuming the server has access to a target’s SNP profile, it aims to infer the target’s membership in the federated dataset. The membership inference attack involves two steps: (i) the server attempts to unshuffle the shared SNPs in the metadata and infer the actual IDs, and (ii) it performs a power analysis (Halimi *et al.* 2021) using the Hamming distance between the target and all individuals in the unshuffled metadata. Therefore, the privacy risk is equivalent to the probability of the server successfully unshuffling the shared SNPs in the metadata, a task that becomes feasible when the server knows the SNP IDs. For example, the server can easily obtain the Minor Allele Frequency (MAF) values and pairwise correlations between SNPs in a reference population based on public knowledge when it knows the set of SNP IDs. The server can perform unshuffling using a greedy algorithm, as suggested in *PPKI*. Notably, the server’s ability to unshuffle largely depends on the knowledge of SNP IDs. *TDS* enables researchers to conceal SNP IDs from the server without compromising utility, significantly restricting the server’s ability to unshuffle. Moreover, even if the server obtains the SNP IDs in one iteration (meaning it can match the unordered SNP sequence shared by the researchers with a target’s SNP sequence), it only affects this iteration. With the limited number of SNPs shared in one iteration, the likelihood of a successful membership inference attack is low. Furthermore, *TDS* can directly adopt the techniques proposed in *PPKI*, such as using synthetic SNPs to enhance privacy by reducing the power of unshuffling.

In addition, when researchers iteratively share partial data, the server may discover the linkage among the SNPs shared throughout the iterative process and use that linkage to match the unordered SNP sequence. To mitigate the privacy risk due to the iterative process, researchers can select SNPs from different chromosomes for each iteration to eliminate the linkage among SNPs in different iterations. Through these privacy-preserving strategies, *TDS* enables efficient collaborative analysis while minimizing the potential privacy risks.

## 4 Results

### 4.1 Test datasets

We use two real-world genome datasets from 1000 Genome Project (1000 Genomes Project Consortium *et al.* 2015) and OpenSNP (Greshake *et al.* 2014).

For *1000genome* datasets, we perform basic quality control steps, including excluding SNPs and individuals with missing values, the removal of SNPs with a MAF below 0.1, and eliminating related samples. Subsequently, we randomly select 9423 SNPs for 2400 individuals utilizing Plink 2.0 (Chang *et al.* 2015). We employ the Genetic Complex Trait Analysis (GCTA) (Yang *et al.* 2011) to simulate the phenotype vectors across five distinct scenarios, each characterized by a different level of between-study heterogeneity and/or skewed phenotype distribution:

*Scenario 1 (no between-study heterogeneity)*: Both the case and control groups in each local dataset comprise 600 individuals.

*Scenario 2 (mild between-study heterogeneity)*: Researcher A’s dataset includes 450 individuals in the case group and 750 in the control group, whereas Researcher B’s dataset contains 750 individuals in the case group and 450 in the control group.

*Scenario 3 (significant imbalance)*: Researcher A’s dataset has a case-to-control ratio of 300:900, while Researcher B’s dataset shows a reversed ratio of 900:300.

*Scenario 4 (highly skewed phenotype distribution)*: Each local dataset comprises 300 individuals in the case group and 900 individuals in the control group.

*Scenario 5 (slight skewness in phenotype distribution and mild heterogeneity)*: While Researcher A maintains a balanced dataset (case-to-control ratio of 600:600), Researcher B’s dataset is imbalanced, with a case-to-control ratio of 300:900.

For the *OpenSNP* dataset, we select 28 976 SNPs on chromosome 15 from 800 individuals, using eye color as the phenotype. The case and control datasets are evenly divided between the two researchers.

### 4.2 Performance analysis

*TDS* comprises two distinct phases. *Phase 1* is centered on researchers identifying insignificant associations within their local datasets to enhance efficiency. In contrast, *Phase 2* focuses on enhancing utility, i.e. detecting significant associations, by facilitating an iterative sharing of partial data with the server.

We commence by evaluating *TDS*’s performance and draw comparisons to *GWAR* (Dimou *et al.* 2017), a publicly available and efficient tool for meta-analysis of GWAS. Among the array of methods offered by *GWAR*, we opted for the Cochran-Armitage Trend Test (CATT) (additive) method. CATT is a prevalent test for GWAS association analyses and does not rely on the assumption of Hardy–Weinberg equilibrium (HWE). The empirical analysis demonstrated that CATT offers potent results and outperforms other tests regarding efficiency. To accommodate between-study heterogeneity, we implemented a random-effects model for the meta-analysis (Cantor *et al.* 2010). Subsequently, we examine the trade-offs between privacy and utility by adjusting parameters such as thresholds (*μ*), sample size (*K*), and the number of iterations *T*. The ground truth significance labels are derived using the entire dataset, with a chosen significance threshold of 0.005. We executed each experiment five times, reporting the average of the results.

**Figure 2.**
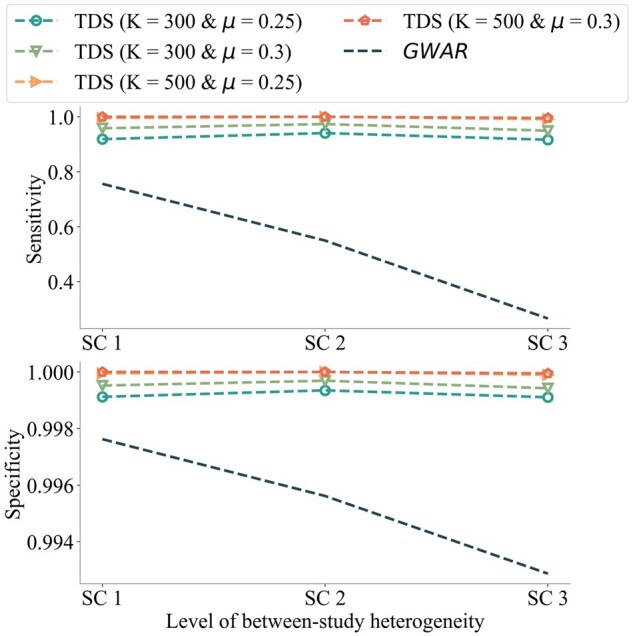
Performance comparison of *TDS* versus *GWAR* under the first three scenarios on *1000genome*. Top: sensitivity. Bottom: specificity. Each scenario has a different level of between-study heterogeneity. We use different *K* and *μ* for each dashed *TDS* line. We set the maximal iterations at nine and report the best performance out of the nine iterations.

The performance of *TDS* and *GWAR* in terms of successful association identification, as measured by sensitivity (the proportion of significant associations correctly identified out of all significant associations) and specificity (the proportion of insignificant associations correctly identified out of all insignificant associations), are depicted in Fig. 2 for the first three scenarios. The results of the proposed method under skewed phenotype distribution and data heterogeneity are presented in Table 1. From Fig. 2 and Table 1, we observe that *TDS* consistently surpasses meta-analysis in performance and can accurately detect true positives. Notably, while the power of *GWAR* (Dimou *et al.* 2017) to identify significant associations dramatically diminishes with increasing heterogeneity and skewness of phenotype distribution, our proposed method continues to achieve high sensitivity. Supplementary Table S1 and Fig. S1 effectively illustrate the trade-off between efficiency and utility. Notably, when a threshold of 0.1 is employed in one scenario, we successfully identify 7406 (78%) insignificant associations without missing any significant associations. Even under the most challenging conditions, such as when μ=0.3 in *Scenario 5*, our method effectively filters 22% of associations during *Phase 1*. It is important to highlight that opting for larger thresholds (μ=0.15) guarantees that no significant SNPs are excluded (with the exception of *Scenario 3*). However, it is not recommended to select an excessively high threshold (μ≫0.3), as it categorizes most of the tested SNPs as “significant” in *Phase 1*, leading to increased runtime in *Phase 2*.

**Table 1.** Performance comparison of *TDS* versus *GWAR* on *1000genome* when the phenotype distribution is skewed (Sens. refers to Sensitivity, while Spec. refers to Specificity).

Method	Scenario 1		Scenario 4		Scenario 5	
	Sens.	Spec.	Sens.	Spec.	Sens.	Spec.
*TDS* (*K *=* *500, μ=0.3)	1	1	0.99	0.999	0.99	0.99
*TDS* (*K *=* *500, μ=0.25)	0.99	0.999	0.99	0.999	0.99	0.99
*TDS* (*K *=* *300, μ=0.3)	0.96	0.999	0.95	0.998	0.98	0.99
*TDS* (*K *=* *300, μ=0.25)	0.92	0.999	0.92	0.997	0.97	0.98
*GWAR* (Dimou *et al.* 2017)	0.76	0.997	0.79	0.993	0.72	0.93

We proceed to examine the trade-off between privacy and utility in *Phase 2*. Supplementary Fig. S2 presents the sensitivity and specificity results during *Phase 2* across each iteration with variations in the number of individuals in each batch and thresholds. Note that the associations detected in *Phase 1* are not included. Interestingly, the performances, measured by sensitivity and specificity rates, of even-numbered iterations surpass those of the odd-numbered ones due to our tie-breaking strategy. When determining the significance label of a SNP after several iterations, we consistently lean towards labeling it as “insignificant” in cases of ties between ‘significant” and “insignificant” (generally observed in even-numbered iterations), a strategy aimed at controlling the false positive rate. *TDS* achieves high sensitivity and specificity across all five scenarios, particularly in *Scenario 5*, which closely simulates real-world settings. In *Scenario 5*, by uniformly selecting 300 and 500 individuals in each iteration, ∼43% and 65% of the entire dataset is employed after two iterations, respectively. Therefore, our method necessitates sharing fewer individuals through the iterative process while achieving high utility. Notably, it is trivial to phrase the proposed method with Bernoulli Sampling, which further amplifies privacy via sampling (Li *et al.* 2012, Ebadi *et al.* 2016).

In summary, experimental results from simulated scenarios suggest our method surpasses meta-analysis in its robustness for identifying correct associations within heterogeneous data. Although the Bonferroni approximation accounts for the family-wise error rate, it tends to be excessively conservative in identifying significant associations when only a few samples are available. The experiments demonstrate that implementing a relatively large threshold *μ* (e.g. 0.3) enhances both efficiency and utility. While a small sample size (*K *=* *300) can achieve high utility, performance can be further amplified by using 500 samples in each iteration. The incurred privacy loss is offset by running fewer iterations. During the experiments, it was noted that *TDS* attained optimal performance at the fourth iterations in *Scenarios 2* and *3*, and at the second iteration in *Scenarios 4* and *5*, respectively, when using a relatively large sample size (e.g. 500) and threshold (e.g. 0.3).

Having highlighted the merits of the proposed method, we proceed to evaluate *TDS* using the *OpenSNP* dataset with real phenotypes. Table 2 reveals that, when applying μ=0.25, our method successfully detects 14 619 (44%) insignificant associations in *Phase 1*. With only two iterations, we are able to identify the majority of significant associations.

**Table 2.** Performance comparison of *TDS* versus *GWAR* on *OpenSNP* dataset (Sens. refers to Sensitivity, while Spec. refers to Specificity).

Method	Overall		*Phase 1*		*Phase 1*
	Sens.	Spec.	Sens.	Spec.	FN (TN)
*TDS* (*K *=* *300, μ=0.3, *T *=* *2)	0.999	0.999	1	1	0 (12837)
*TDS* (*K *=* *300, μ=0.25, *T *=* *2)	0.999	0.999	0.999	0.999	0 (14619)
*TDS* (*K *=* *200, μ=0.3, *T *=* *5)	0.996	0.999	0.996	0.999	0 (12837)
*GWAR* (Dimou *et al.* 2017)	0.732	0.992			

We then show the efficiency of *TDS* and investigate how the runtime of *TDS* changes with varying numbers of samples and SNPs (see Supplementary Table S2 and Fig. S3 for details). Compared to sPLINK (Nasirigerdeh *et al.* 2022), *TDS* is around 1.5 times faster (43.5 min versus 65 min for 400K SNPs) without multi-threading and a larger sample size (6000 versus 5823). Moreover, the results show that the runtime increases nearly linearly with the number of SNPs, which can be reduced by using parallel computation techniques. As the sample size grows, runtime does not change much.

## 5 Discussion and conclusion

In this study, we introduce *TDS*, a novel two-phase strategy designed to mitigate the computational challenges inherent in existing SMC and HE solutions. *Phase 1* facilitates local computations in plaintext, thus minimizing the need for inter-site communication and encryption/decryption operations. On the other hand, *Phase 2* adopts an iterative principle to decrease data sharing and consequently reduce privacy risks. The primary objective of our method is to improve the feasibility and efficacy of collaborative GWAS, thereby expediting genomics research while adhering to safety and privacy regulations. We want to emphasize that our intention is not to propose alternative genomic analytics methods. Instead, our goal is to manage privacy risks to ensure they remain within the acceptance boundaries of many institutions, such as those stipulated by the National Institutes of Health (NIH) Genomic Data Sharing (GDS) policy.

In Section 4, we have demonstrated that employing a more relaxed threshold (for instance, 0.3 compared to the Bonferroni correction) enables the identification of over half of the insignificant SNPs using only the local dataset. It is critical to emphasize, however, that the effectiveness of *Phase 1* is contingent on the size of the dataset. Larger sample sizes produce more accurate results. Furthermore, the effectiveness also depends on the degree of heterogeneity in the phenotype distribution. In an extreme scenario where only control (or case) samples are present across all researchers, *Phase 1* becomes non-applicable. However, *Phase 2* can still operate effectively when the pooled datasets contain more than one group of samples. This is possible when some hospitals possess only case samples while others have control samples.

Currently, *TDS* focuses on logistic regression tests but can be expanded to accommodate quality control steps used in a GWAS. These steps include the computations of MAF, HWE, Linkage Disequilibrium (LD), and relatedness identification. Federated computation of MAF, HWE, and LD is anticipated to be straightforward, as they are based on allele frequencies and can be attained in *Phase 1*. Moreover, a recent framework, *INK* (Wang *et al.* 2023), devised for federated kinship identification, can be easily incorporated into *TDS* as it employs a similar system model. Addressing population stratification presents greater challenges in the federated setting, as merging two or more datasets with completely homogeneous populations in isolation can inadvertently introduce bias due to underlying cross-correlations. The application of PCA, a common method for addressing population stratification, is also not straightforward given the privacy requirements. Furthermore, federated PCA algorithms may require more data exchange and numerous iterations to converge on final eigenvectors, giving rise to security and runtime concerns. Developing an efficient federated PCA algorithm to handle both horizontally and vertically partitioned data will be a subject of our future research.

In this article, our proposed framework aims to mitigate general privacy risks without specifying particular attacks or delving into intricate threat models. We prioritize providing a practical and flexible solution that can be applied broadly in scenarios where the threat landscape may vary. Future research endeavors may delve deeper into specific privacy threats, consider more stringent threat models, and explore how to tailor defenses against particular types of attacks while maintaining the overarching privacy principles.

## Supplementary Material

btad639_Supplementary_Data

## Data Availability

The code and datasets are available at https://github.com/amioamo/TDS/tree/main.
