# Supplementary material for: Privacy-preserving federated genome-wide association studies via dynamic sampling
Source: Bioinformatics. 2023 Oct 19;39(10):btad639. doi: 10.1093/bioinformatics/btad639 (PMC10612407; doi:10.1093/bioinformatics/btad639)
Supplement: btad639_Supplementary_Data [file btad639_supplementary_data.pdf]

Supplementary Material for "Privacy-Preserving Federated Genome-wide Association Studies via Dynamic Sampling"

1. TWO-STEP DYNAMIC SAMPLING (TDS) FRAMEWORK

Algorithm S1. Two-Step Dynamic Sampling Federated GWAS

Input: Individuals genotype arrays, $\boldsymbol{D}^A$ and $\boldsymbol{D}^B$, and phenotype vectors, $\boldsymbol{p}^A$ and $\boldsymbol{p}^B$, from researchers A and B, significance threshold μ, data size K

Output: Significance labels of SNPs

Phase 1: Detecting local insignificant associations

for $r \in \{A, B\}$ **do**

  Researcher r performs logistic regression tests with D^r and $\boldsymbol{p}^r$ and obtains p-values for each SNP in I

  $INSIG\ I^r = \{SNP_i | p\text{-value of } SNP_i > \mu, SNP_i \in I\}$

Researcher A sends $INSIGI^A$ to Researcher B

Researcher B sends back $INSIG\ I = INSIG\ I^A \cap INSIG\ I^B$ to A

Phase 2: Detecting federated significant associations

Initialize $I = I \setminus INSIG\ I$ and $SIG = \emptyset$

$T = 1$

while stop condition is not satisfied **do**

  Sync Stage

  A and B decide the partition of I, partition the SNPs across the batches, i.e., $I = \cup_{b \in \boldsymbol{b}} I_{b,T}$, $I_{b,T} = \{SNP_{b_i} | i = 1, 2, \cdots, m_{b,T}\}$ and the permutation seed vector $\boldsymbol{U}_T$

  for $r \in \{A, B\}$ **do**

    for each batch b **do**

      $\boldsymbol{D}'^r_{b,T}$ ($\boldsymbol{p}'^r_{b,T}$) $\leftarrow$ K individuals selected uniformly at random from $\boldsymbol{D}^r_{b,T}$ (correspondingly $\boldsymbol{p}^r_{b,T}$)

      Permute the SNPs (columns) of $\boldsymbol{D}'^r_{b,T}$ with $U_{b,T}$ for each batch

      Shuffle the K individuals (rows) of $\boldsymbol{D}'^r_{b,T}$ and $\boldsymbol{p}'^r_{b,T}$ for each batch

      Remove the SNP IDs and sample identifiers (sample IDs) from $\boldsymbol{D}^r_{b,T}$ and $\boldsymbol{p}^r_{b,T}$ for each batch giving $\tilde{\boldsymbol{D}}^r_{b,T}$ and $\tilde{\boldsymbol{p}}^r_{b,T}$

  Outsourcing Stage

  A and B sends $\{\tilde{\boldsymbol{D}}_{1,T} \oplus \tilde{\boldsymbol{p}}_{1,T}, \tilde{\boldsymbol{D}}_{2,T} \oplus \tilde{\boldsymbol{p}}_{2,T}, \cdots, \tilde{\boldsymbol{D}}_{b,T} \oplus \tilde{\boldsymbol{p}}_{b,T}\}$ to Server

  Server computes the logistic regression tests on each batch and returns p-values to A and B

  for $r \in \{A, B\}$ **do**

    Recover the order of p-values of all the batches based on $\boldsymbol{U}_T$

    Obtain the significance level for all the SNPs, $\boldsymbol{l}_T$

    Update the significance level of SNP_i in I with $l^*_i = MODE\ (l_{i,1}, l_{i,2}, \cdots, l_{i,T})$

    $SIG = SIG \cup \{SNP_i | l^*_i = 1\} \setminus \{SNP_i | l^*_i = 0\}$

  $T = T + 1$

2. IMPACT OF PARAMETERS ON EFFICIENCY, UTILITY, AND PRIVACY

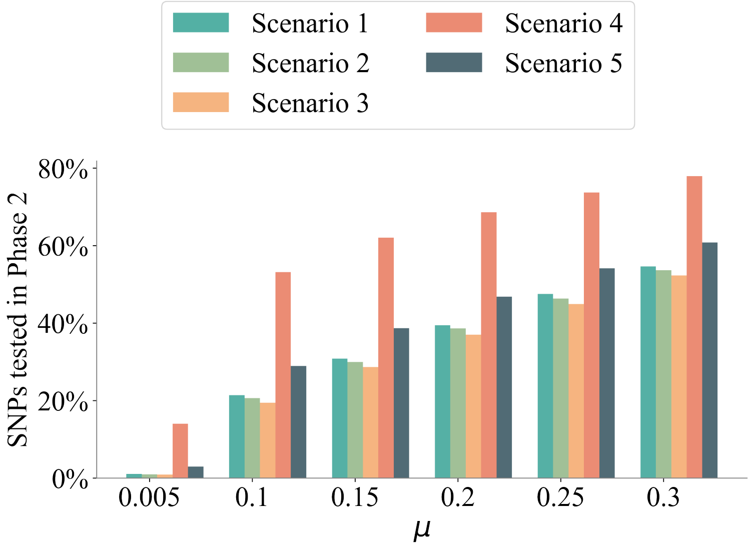

Fig. S1. Impact of different thresholds (μ) used in *Phase 1* on the number of SNPs to be shared in *Phase 2* in all five scenarios.

Table S1. Number of false negatives and true negatives (in parenthesis) detected in *Phase 1* on *1000genome*.

	$\mu = 0.005$	$\mu = 0.1$	$\mu = 0.15$	$\mu = 0.2$	$\mu = 0.25$	$\mu = 0.3$
Scenario 1	58 (9266)	0 (7406)	0 (6516)	0 (5703)	0 (4941)	0 (4274)
Scenario 2	66 (9266)	0 (7476)	0 (6596)	0 (5780)	0 (5058)	0 (4364)
Scenario 3	73 (9265)	7 (7582)	2 (6717)	1 (5934)	0 (5188)	0 (4493)
Scenario 4	162 (8978)	1 (6697)	0 (5774)	0 (5011)	0 (4318)	0 (3691)
Scenario 5	848 (7250)	1 (4410)	0 (3574)	0 (2959)	0 (2478)	0 (2077)

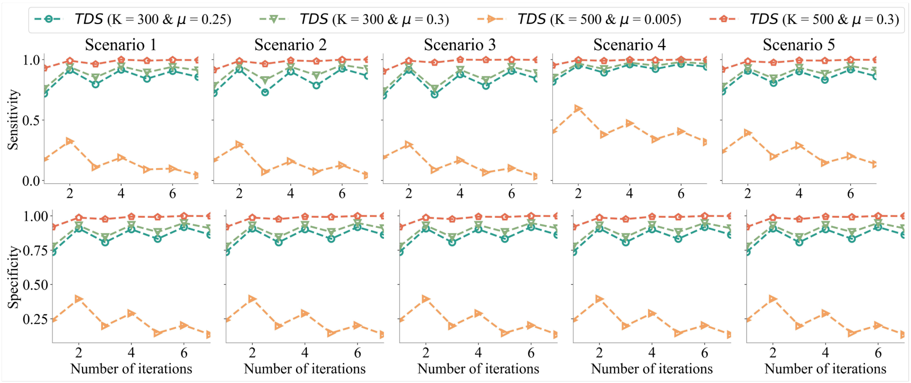

Fig. S2. Impact of different data size (K) and threshold (μ) in all five scenarios. Upper: sensitivity. Lower: specificity. Insignificant SNPs detected in *Phase 1* are not included.

3. RUNTIME ANALYSIS

We simulated a datasets with 4000 individuals (2000 each for case and control grouop) and 400K SNPs using Plink 1.9 [1]. The experimental setup used for measuring the runtime of *TDS* is given in Table S2.

Table S2. The experimental setup used for measuring the runtime of *TDS*

Description	# of researchers	# of Sample (case:control=1:1)	# of SNPs	Parameters
Varying sample sizes	2	1K, 2K, 4K	100K	$\mu = 0.3, K = 300, T = 7$
Varying numbers of SNPs	2	2K	100K, 200K, 400K	$\mu = 0.3, K = 300, T = 7$

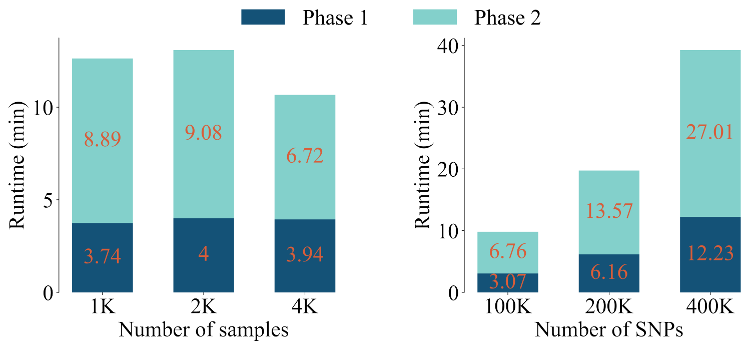

Fig. S3. Runtime of *TDS* with varying number of samples (left) and SNPs (right)

REFERENCES

1. C. C. Chang, C. C. Chow, L. C. Tellier, S. Vattikuti, S. M. Purcell, and J. J. Lee, “Second-generation plink: rising to the challenge of larger and richer datasets,” Gigascience **4** (2015).
